# Optimized Conformal Total Body Irradiation with VMAT Using a Linear-Accelerator-Based Radiosurgery Treatment System in Comparison to the Golden Standard Helical TomoTherapy

**DOI:** 10.3390/cancers15174220

**Published:** 2023-08-23

**Authors:** Mümtaz Köksal, Oğuzhan Özkan, Tobias Holderried, Annkristin Heine, Peter Brossart, Ahmed Gawish, Davide Scafa, Gustavo R. Sarria, Christina Leitzen, Leonard C. Schmeel, Thomas Müdder

**Affiliations:** 1 Department of Radiation Oncology, University Hospital of Bonn, 53127 Bonn, Germany; 2 Department of Internal Medicine—Oncology, Hematology and Rheumatology, University Hospital of Bonn, 53127 Bonn, Germanypeter.brossart@ukbonn.de (P.B.); 3 Department of Radiation Oncology, University Hospital of Marburg, 35043 Marburg, Germany

**Keywords:** total body irradiation, helical tomotherapy, volumetric modulated arc therapy, stereotactic radiosurgery

## Abstract

**Simple Summary:**

Total body irradiation (TBI) as a component of conditioning regimens prior to hematopoietic stem cell transplantation is performed differently by various radiotherapy centers. In general, conventional methods are predominantly applied. With the introduction of advanced irradiation techniques such as Helical TomoTherapy (HT) and Volumetric Modulated Arc Therapy (VMAT), dose-optimized total body irradiation could be realized with good target volume coverage and adequate dose reduction in organs at risk (OAR). This requires a well-coordinated team and suitable equipment capabilities. Centers similar to ours can offer HT treatment as a gold standard for highly conformal TBI. Based on the limited replacement equipment availability in case of HT device failure, we developed a method carrying out highly conformal VMAT irradiation with a linear accelerator originally suitable for radiosurgery or stereotactic radiotherapy. Using a dosimetric comparison between both modalities, we examined whether VMAT—even with linear accelerator radiosurgery technology—is capable of performing optimized conformal TBI.

**Abstract:**

Modern irradiation techniques for optimized conformal TBI can be realized by Helical Tomotherapy (HT) or Volumetric Modulated Arc Therapy (VMAT), depending on the availability of suitable specialized equipment. In this dosimetric planning study, we compared both modalities and addressed the question of whether VMAT with small field sizes is also suitable as a backup in case of HT equipment malfunctions. For this purpose, we retrospectively used planning computed tomography (CT) data from 10 patients treated with HT with a total dose of 8 Gy (n = 5) or 12 Gy (n = 5) for treatment planning for VMAT with a small field size (36 × 22 cm). The target volume coverage, dose homogeneity at target volume, and dose reduction in organs at risk (OAR) (lungs, kidneys, lenses) were analyzed and compared. One patient was irradiated with both modalities due to a device failure of the HT equipment during the study, which facilitated a comparison in a real clinical setting. The findings indicate that in addition to a higher mean dose to the lenses in the 12 Gy group for VMAT and a better dose homogeneity in the target volume for HT, comparably good and adequate target dose coverage and dose reduction in the other OAR could be achieved for both modalities, with significantly longer treatment times for VMAT. In conclusion, after appropriate optimization of the treatment times, VMAT using linear accelerator radiosurgery technology can be used both as a backup in addition to HT and in clinical routines to perform optimized conformal TBI.

## 1. Introduction

Allogeneic hematopoietic stem cell transplantation is used for the therapy of various hematological diseases [1]. Prior to successful transplantation, the patient is conditioned to eliminate diseased/malignant cells, and their immune system is suppressed to prevent rejection of the transplant. In addition to chemotherapeutic agents, total body irradiation (TBI) is an important component of different conditioning regimens. The advantage of the TBI treatment protocol is its dose application without interference from blood supply or pharmacokinetic factors [2], and it is predominantly used in conditioning regimens for patients with high-risk and recurrent acute myeloid leukemia (AML) and acute lymphoblastic leukemia (ALL) [2,3].

The goal of TBI is to irradiate a large volume homogeneously with an adequate dose while ensuring minimal toxicity to organs at risk (OAR) so that the therapeutic effect of TBI is not counteracted by therapy-associated complications. For example, a randomized trial by Clift et al. in which two groups were irradiated with a total dose of 15.75 Gy or 12 Gy with seven and six fractions, respectively, showed no difference in overall survival. Although the probability of relapse was significantly lower in the 15.75 Gy group, the non-relapse mortality was higher in this group [4].

Since conventional TBI techniques have limited options for achieving dose reduction in OAR, elaborately manufactured blocks are used for organ shielding. Lung shielding is the most commonly used form of organ shielding [3] because lung toxicity is one of the dose-limiting factors, and irradiation-induced pneumonitis is one of the major contributors to therapy-associated morbidity and mortality in TBI [5]. For this reason, most centers try not to exceed a median lung dose of 8–10 Gy [2].

Technically, homogeneous irradiation of such a large volume is challenging. Older conventional methods are planned two-dimensionally (2D) with reference points on the body surface and use an extended source-to-skin distance (SSD) so that the entire body is covered by the radiation field. Not only patient positioning (standing, sitting, lying) but also gantry position can vary, with a beam path either anterior–posterior/posterior–anterior or lateral [6]. Some conventional methods use special devices, such as a translational couch, which moves the patient through the radiation field and thus requires a shorter SSD [7].

A long irradiation time, in combination with mostly uncomfortable patient positioning, makes conventional methods burdensome, especially for patients who are in poor general condition. In addition, 2D-planned dose distribution in conventional TBI is not accurate and does not precisely reflect the actual dose distribution in a three-dimensional (3D) body. In computed tomography (CT)-based on 3D treatment planning for conventional TBI with lateral beam direction, Hui et al. showed significant dose heterogeneities with overdoses in the lung and underdoses in the target volume [8]. In addition, inadequate organ shielding might reduce the dose of OAR; however, it could simultaneously lead to underdoses located immediately behind the shield and, thus, to an increased relapse rate [9].

The introduction of modern therapy devices enabled the development of targeted and individualized therapy concepts, also for TBI. Helical Tomotherapy (HT), a novel intensity-modulated radiotherapy technique with integrated advanced image guidance, was introduced in the 1990s [10]. CT-based 3D treatment planning, advanced image guidance, and a helical geometry of dose delivery in one continuous beam facilitate targeted conformal irradiation with dose optimization. The feasibility of this modality to TBI was demonstrated by several groups [11,12]. Homogenous target volume coverage in combination with acceptable toxicity through dose reduction in OAR was demonstrated in various publications [13,14,15]. Even complex volumes, such as the entire thoracic wall with close proximity to the lung, can be adequately irradiated with this modality resulting in good lung sparing [16,17]. On the other hand, optimized conformal intensity-modulated TBI was also developed using alternatively linear accelerators (Linac) [18]. With volumetric modulated arc therapy (VMAT), a Linac also offers a radiation source rotating around the patient. Beams can simultaneously be shaped to the target volume using a multi-leaf collimator (MLC), while the dose rate and rotation speed can be adjusted at the same time, thereby enabling highly conformal irradiation [19].

With the flexibility of modern techniques to optimize irradiation to complex target volumes, a further step toward targeted irradiation has been taken. Instead of irradiating the entire body, it is also possible to target the bone marrow with or without large lymphatic drainage pathways [11,12].

In contrast to the widely-used Linac, only a few centers are equipped with an HT in the first place. Even fewer centers have a second HT as a backup. However, according to the Guidelines from the International Lymphoma Radiation Oncology Group (ILROG), backup equipment should be available to switch to another machine in case of unexpected machine breakdown to prevent treatment failure [2]. Therefore, we developed a method for TBI with VMAT as a backup. Being furnished with a Linac suitable for radiosurgery as a replacement option, we specifically address the question of whether VMAT is suitable for optimized conformal TBI, even with the technical limitation of smaller field sizes which are rather suitable for radiosurgery or stereotactic radiotherapy.

## 2. Materials and Methods

### 2.1. Patient Characteristics

Our study retrospectively included 10 patients who received TBI at HT between July 2020 and February 2023 as part of conditioning before allogeneic stem cell transplantation. Six patients had a diagnosis of T/B-ALL (T/B-cell acute lymphoblastic leukemia), while two patients demonstrated relapsed AML. One patient displayed mixed-phenotype acute leukemia (M-PAL), and another patient suffered from mast cell leukemia (MCL). The patients ranged in age from 15 to 62 years, their body height varied between 152.5 and 190 cm, and their body width ranged between 40.8 and 53.3 cm. Five patients received a total dose of 8 Gy, and the remaining five patients received 12 Gy. The total dose was applied to all patients in 2 × 2 Gy per day for 2 or 3 consecutive days, and a period of at least 6 h comprised the time interval between fractions per day.

### 2.2. Treatment Planning

Since TomoTherapy^®^ Hi-ART II (Accuray Incorporated, Sunnyvale, CA, USA) and the TrueBeam™ STx (Varian Medical Systems, Inc., Palo Alto, CA, USA) have limited couch movement (135 cm for TomoTherapy^®^ and 80 cm for TrueBeam STX^®^ with a BrainLab^®^ Robotics 6d couch), two separate irradiation plans had been developed for treatment. One for the upper body (from head to mid-thigh) and one for the lower extremities (from feet to proximal thighs). For this purpose, two planning CT scans, each with a 5 mm slice thickness, were obtained in the head-first orientation for the upper body plan and in the feet-first orientation for the leg plan. Due to the absence of OAR in the leg plan, we combined a modulated technique for the upper body plan and a static approach to the leg plan. For irradiating lower extremities, an anterior–posterior/posterior–anterior technique was used for VMAT, whereas a conventional 4-field box technique was used for HT [20]. For patient fixation, thermoplastic four-point masks for the head–neck region were used together with vacuum mattresses to keep patient position constant between the planning and treatment sessions. For irradiation planning, planning CT images were transferred to the appropriate treatment planning system (TPS) (TomoTherapy^®^ HiART II planning software, version 5.1.1.6or Eclipse™ Treatment Planning System, version 13). After irradiation planning and optimization, the upper body plan was merged with the leg plan. For contouring, the planning target volume (PTV) is defined as the body’s outer boundary minus 3 mm and without OAR (lens left/right, lung left/right, kidney left/right). OAR were contoured without a margin. We compared calculated plans of all patients, focusing on PTV dose coverage and dose exposure/reduction for OARs. Dose specifications for the target volume were predefined for patients based on a percentage of the prescribed dose (see Table 1). All values apply to patients with clinically healthy OAR. Dose exposure (Dmean) for lungs and kidneys was defined as optimal at 7–8 Gy and acceptable at 9 Gy.

**Figure 1:**
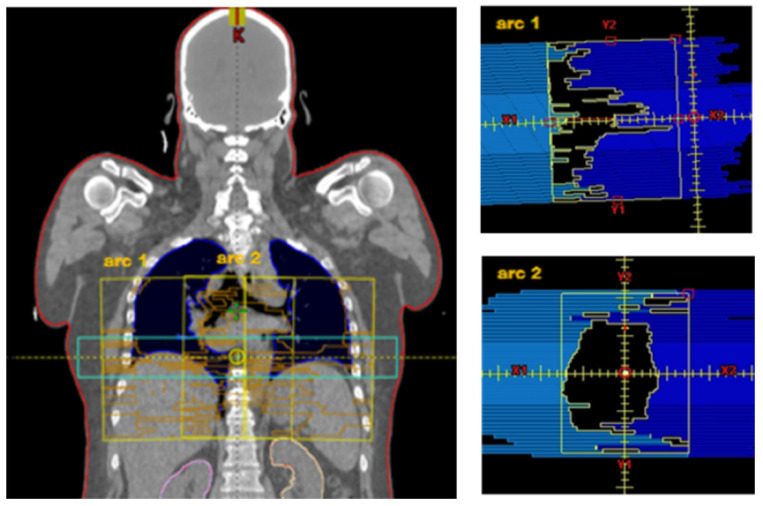
Left: VMAT fields (yellow) for 3 arcs with mediolateral offset: arc1 −18 cm to −2 cm, arc2. −7.5 cm to 7.5 cm, arc3 3.0 cm to 18.5 cm. For comparison purposes, see the HT field in light blue. Right: arc1 with collimator 5° in x-direction asymmetric: −18.0 cm to −2.0 cm. arc2 with collimator 0° in x-direction symmetrical: −7.5 cm to 7.5 cm. Body surface as target volume (PTV) is visible in red.

The VMAT method was developed for a TrueBeam™ STx (Varian Medical Systems, Inc., Palo Alto, CA, USA) with an HD120^®^ MLC. The HD120^®^ MLC is equipped with 2 opposing rows of 60 leaves. The central 32 leaves have a width of 2.5 mm, and the outer 28 leaves measure 5 mm, which results in a field width of 22 cm in y-direction. For a VMAT rotation, field size in x-direction measures 30 cm. This results in a maximum field size for a rotation of 30 × 22 cm, with which the entire PTV apparently cannot be well covered. However, the HD120 MLC can be set up asymmetrically in x-direction. As an example, for a field size of 36 × 22 cm, a total of three full rotations (short arc) with field sizes for arc1 from −18 cm to −2 cm, for arc2 from −7.5 cm to 7.5 cm and for arc3 from 2 cm to 18 cm are needed. The collimator angles for those three arcs are 355°, 0°, and 5° (see Figure 1).

**Figure 2:**
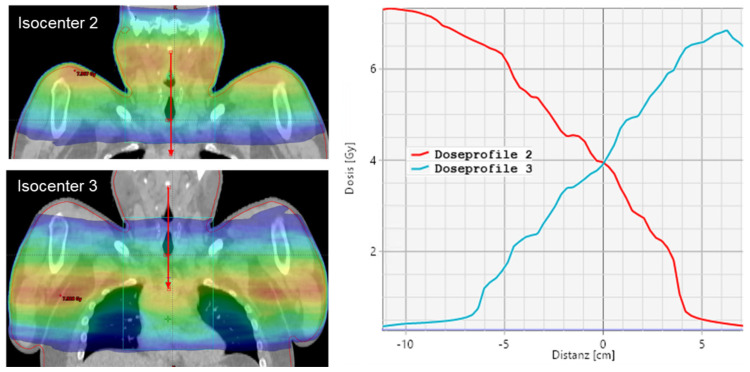
Dose profile for isocenter 2 and 3 for a VMAT plan.

An overlap of the arcs of two isocenters x and x + 1 is between 4 and 8 cm for the upper body, depending on patient width and the degree of modulation for protection of lungs and kidneys. With this field configuration, a slightly decreasing dose gradient into the consecutive isocenter is obtained (see Figure 2).

**Figure 3:**
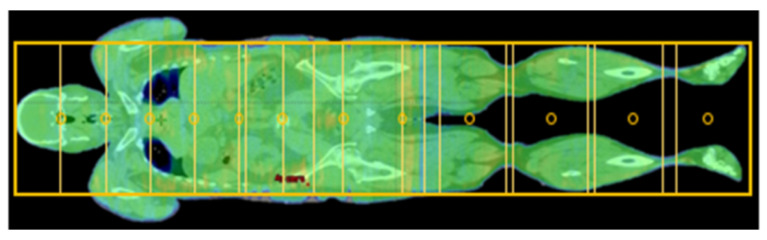
Isocenters (circles) and field configuration (boxes) for a VMAT plan for an adult patient.

A robust, comprehensive plan against craniocaudal displacements is ensured, in addition to good coverage of the target volume with simultaneous good protection of organs at risk. An overlap area of two isocenters for the legs counts only 2 cm to 4 cm due to small width and weak modulation. This results, in general, between 10 to 14 isocenters, each with 3 arcs for adult patients (see Figure 3).

For position verification, image guidance for VMAT was performed using cone beam CT (CBCT) before each IC setting. For HT, integrated megavoltage CT (MVCT) was performed before the start of upper body irradiation and leg irradiation, respectively. After the application of the upper body irradiation, the patient had to be rotated 180° from the head-first position to the feet-first position for both modalities. Quality assurance was performed by dosimetry on the flat panel and multichannel diodes for the first fraction.

For assessing plan quality, the following dose parameters were determined for both irradiation plans: for the target volume PTV, D95%, D98%, D50%, and D2% were determined, and the homogeneity index (HI) was calculated.

According to ICRU83, we used the following formula for this purpose:

$$\mathrm{HI}=\frac{D2\%-D98\%}{D50\%}.$$

The mean dose (Dmean) was determined for various OAR, and the values for both plans were tested for significant differences using an unpaired *t*-test. Since the leg plans were irradiated using a static technique in both modalities, only the beam-on time of the upper body plans was compared. Due to an equipment failure of the HT, which occurred during the treatment of patient 10, switching to a VMAT plan for 2 fractions allowed the comparison between VMAT and HT for the same patient in a real clinical setting. The total treatment time for two fractions, including imaging, patient positioning and rotation, and beam-on time, was registered and compared.

## 3. Results

Both VMAT and HT modalities show adequate target volume coverage with comparable dose distribution at the target volume. The differences are at non-significant levels for both the 8 Gy and 12 Gy groups, tending to higher doses for D2% and D50% and lower doses for D98% and D95% in VMAT compared to HT (Table 2). The homogeneity index for both dose groups is better in HT (Table 2).

Overall, a dose reduction has been achieved in OAR within the range of the actual guidelines according to the current literature [2,21]. Although differences for the Dmean of the lungs and kidneys are at non-significant levels, slightly higher values for the VMAT can be observed in both dose groups (Table 3). The average Dmean lung left/right values for VMAT versus HT in the 8 Gy group are 6.58/6.52 Gy versus 6.44/6.42 Gy, and in the 12 Gy group, the values are 8.47/8.59 Gy versus 7.99/7.99 Gy. The Dmean lung range is below 9.4 Gy. The average Dmean values at the kidneys left/right are not significantly different for VMAT and HT in the 8 Gy group with 6.51/6.53 Gy versus 6.35/6.31 Gy and in the 12 Gy group with 8.85/8.27 Gy versus 8.35/8.27 Gy. For the left/right lens, a significantly high Dmean was observed in the 12 Gy group for the VMAT with 4.14/4.01 Gy versus 2.59/2.58 Gy for the HT. In the 8 Gy group, the lens doses for the VMAT were not significantly higher.

**Figure 4:**
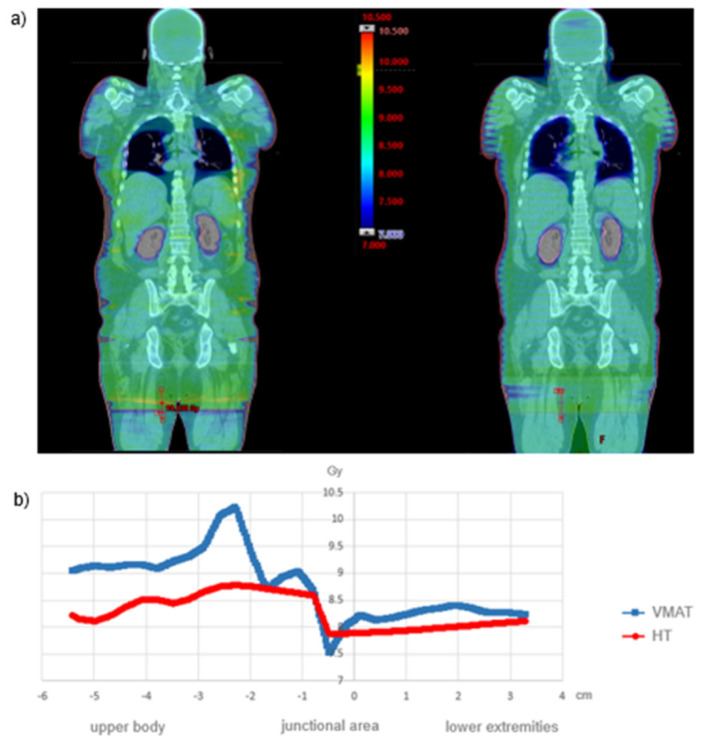
(**a**) Dose distribution for VMAT and HT in the coronal plane and (**b**) dose profile in the junctional area in the longitudinal axis.

As can be seen from the exemplary isodose distribution in Figure 4a, near-skin dose decreases were recorded at the lateral field edges due to the restricted field size for both the VMAT and the HT. Nevertheless, due to the multidirectional radiation, a dermal dose of at least 7 Gy was achieved. Considering the high radiation sensitivity of leukemia cells with a D0 value (decimal reduction dose) of 0.74 Gy [22,23], near-skin underdosage is not clinically relevant. Furthermore, the dose profile in the junctional area reveals a more uniform transition for HT. In contrast, VMAT displays a more heterogeneous transition with a focal hotspot (Figure 4b). Since there are no OAR in the junctional area, this circumstance will not have a critical impact on toxicity. 

Using VMAT for the upper body area, the arcs of the two isocentres do overlap, as shown in Figure 3. Depending on the body length of a patient, an overall plan with 5 to 7 isocentres is created for this body region. Thus, entire junctional areas between each single isocentre can be optimized wholly in one plan. According to the physical limits of the linear accelerator, this approach allows a well-modulated single treatment plan. A joint optimization results in a homogeneous dose profile. Especially in comparison to the junctional body/leg area, which is planned independently in a consecutive further step.

In practice, while performing the irradiation of the head, which is the first isocenter, the couch has a value z in the longitudinal direction. Based on this value z, physicist experts calculate longitudinal displacement for the other consecutive isocenters and monitor compliance with the calculated values. CBCT or kV/MV imaging is concurrently used for each isocenter to guarantee prompt position verification. This workflow enables a radiation oncologist’s immediate observation of any clinically relevant intra-fractional patient position irregularity.

Since patient 10 was treated on both devices, a practical comparison is possible, particularly concerning the clinical processes and therapy times. The total therapy time consists of the time of dose application (beam-on time) on the one hand and the patient setup on the other hand. The latter includes the time for image guidance and patient positioning, as well as for patient rotation between the upper body and leg plans. It is noticeable that the total treatment time for one fraction on the VMAT is significantly longer than that of HT (Table 4). The two fractions applied with VMAT lasted 146.8 and 183.4 min, whereas the fractions applied with HT lasted almost half as long, with 77.8 and 82.98 min, respectively. With a duration of approximately 50 min, the beam-on time for the applied 8 Gy lasted significantly longer with VMAT than with HT, which required approximately 30 min. Furthermore, the patient rotation from the head-first position to the feet-first position was also longer with VMAT and lasted up to 20 min, whereas in HT, the rotation lasted 3 to 4 min. The mean beam-on time for all upper body plans in the 8 Gy group was 25.8 min (±6.2 min) with VMAT, which was almost twice as long as the 16.7 min (±1.3 min) with HT and similar for the 12 Gy group with 27.4 vs. 16.7 min (Table 5).

## 4. Discussion

As demonstrated in previous feasibility studies, HT and VMAT are ideally suited for the application of high conformal TBI. Even a Linac with small field sizes, which is intended for stereotactic irradiation, can be used for conformal TBI. We were able to compare radiation plans on both modalities using identical image data. For both modalities, dose coverage in the target volume falls within the criteria stipulated by international guidelines [24]. However, as indicated in previous publications, a more homogeneous dose application is possible with HT [25,26], while adequate sparing of the OAR (lungs, kidneys, lens) is possible in both modalities. Although the lens dose is significantly larger for VMAT in the 12 Gy group, the average Dmean values for the left and right lens of approximately 4 Gy are well below the 12 Gy limit recommended by the ESTRO SIOPE Guidelines for pediatric patients and thus are not clinically relevant [21].

Based on the actual irradiation of one patient on both modalities, we were also able to compare the treatment times. As postulated by Nalichowski et al. [26] and shown by another single-center study [25], the total treatment time with VMAT is disproportionally longer than with HT. A fraction with VMAT lasted between 2.5 and 3 h, whereas a fraction with HT took merely 1.2 h. The main cause lies in the patient setup required for VMAT since CBCT or kv/MV is performed before each IC setting, and the patient must be aligned accordingly if necessary. Although patient rotation also took significantly longer with VMAT, proper practice experience of patient setup could certainly be shortened by 30 min. Total treatment time with VMAT of approximately 2 h should be achievable with specialized, easy-to-use devices for patient positioning. Quyang et al. and Losert et al. presented a special rotation table that easily and accurately rotates the patient by 180° between the upper body and leg plan in approximately 3 min [27,28].

Furthermore, the beam-on time for the VMAT method was almost twice as long as on HT, whereby the beam-on time of VMAT is the sum of all individual arc rotation times, which can have different durations depending on the plan complexity and the given MLC motion speed limitations. Since we had to insert more IC because of the small field size (a total of 8 VMAT IC for the upper body plan), our beam-on time of 27.4 min took longer compared to similar VMAT-TBI methods with lower IC. Tas et al. reported in contrast to our experience of 15.4 min with 3 IC [28]. Springer et al. experienced a VMAT-TBI method with a similar number of IC (9–15), resulting in a total treatment time of 1.5 to 2.0 h, which is comparable to our total treatment time [29,30].

Since HT seems to be a faster and easier way to perform TBI than VMAT, with nearly equivalent target volume coverage in conjunction with dose reduction at OAR, it can justifiably be named the gold standard in highly conformal TBI. Moreover, it may be noted that conformal TBI with VMAT is possible even with small field sizes using a radiosurgery treatment system. Although the sequential dose delivery technique in optimized conformal TBI raises concerns about tumor cells floating in the circulating blood volume not adequately irradiated, Molloy et al. showed that typical treatment times and total body doses are sufficient to effectively eliminate tumor cells in the circulating blood volume [31]. This may also be due to the fact that leukemia cells have a high radiation sensitivity with a D0 of 0.74 Gy [23].

Regardless of these technical advancements, several surveys unveil marked differences between radiation centers in the current delivery of TBI [32,33,34,35,36]. Still, outdated conventional 2D-scheduled irradiation techniques are the most used methods [3,33,37]. Unfortunately, present heterogeneous irradiation techniques used in various centers limit the comparability of their findings and the ability to make a representative statement regarding TBI. Another challenge is that most renowned clinical trials involving the indication of TBI in conditioning are based on older conventional non-intensity-modulated irradiation methods. Promising results obtained from early clinical phase I and II trials will hopefully provide the basis for larger multicenter studies [38,39]. This will require more precise guidelines increasing the comparability of complex conformal TBI methods between various centers even more. It could also boost the motivation of more centers worldwide to apply modern methods and discard the outdated conventional TBI approach.

## 5. Conclusions

In addition to assessing the existing HT technique used as the gold standard for optimized conformal TBI in our radiotherapy center, an acceptable VMAT TBI method with small field sizes could also be developed. The main disadvantage of this VMAT method lies in the relatively long treatment time, which could, however, be reduced after appropriate process optimizations. Finally, VMAT using a linear accelerator suitable for radiosurgery is not only reasonable as a backup plan in cases of HT equipment failure but can also be used in clinical routine practice for optimized conformal TBI. This might be relevant, particularly for radiotherapy centers that do not facilitate Helical TomoTherapy.

## Figures and Tables

**Table 1:** Dose requirements for PTV as % of prescribed dose.

	Optimal	Acceptable
**D98%**	>90%	>87.5%
**D95%**	=100%	=95.0%
**D50%**	<104%	<D106%
**D2%**	<115%	<D120%

**Table 2:** PTV average dose values and HI ± standard deviation (SD) for VMAT and HT in the 8 Gy and 12 Gy groups. Adjusted *p*-value at the 5% significance level for the unpaired *t*-test.

	8 Gy			12 Gy		
	VMAT [Gy]	HT [Gy]	*p*	VMAT [Gy]	HT [Gy]	*p*
**D95%**	7.61 ± 0.33	7.90 ± 0.26	0.63	11.82 ± 0.23	11.96 ± 0.06	0.66
**D98%**	6.96 ± 0.52	7.54 ± 0.38	0.47	11.05 ± 0.26	11.22 ± 0.35	0.77
**D50%**	8.34 ± 0.10	8.29 ± 0.04	0.90	12.64 ± 0.14	12.48 ± 0.08	0.37
**D2%**	8.92 ± 0.25	8.66 ± 0.11	0.43	13.43 ± 0.24	13.03 ± 0.10	0.072
**HI**	0.23 ± 0.08	0.14 ± 0.05		0.20 ± 0.03	0.14 ± 0.03	

**Table 3:** OAR average Dmean ± SD for VMAT and HT in the 8 Gy and 12 Gy groups. Adjusted *p*-value at the 5% significance level for the unpaired *t*-test.

	8 Gy			12 Gy		
	VMAT [Gy]	HT [Gy]	*p*	VMAT [Gy]	HT [Gy]	*p*
Lung left	6.58 ± 0.37	6.44 ± 0.63	0.91	8.47 ± 0.55	7.99 ± 0.51	0.66
Lung right	6.52 ± 0.40	6.42 ± 0.70	0.91	8.59 ± 0.41	7.99 ± 0.54	0.43
Kidney left	6.51 ± 0.40	6.35 ± 0.40	0.91	8.85 ± 0.67	8.35 ± 0.95	0.82
Kidney right	6.53 ± 0.37	6.31 ± 0.42	0.90	8.27 ± 0.45	8.27 ± 1.05	1.0
Lens left	3.02 ± 0.56	2.40 ± 0.41	0.47	4.14 ± 0.62	2.59 ± 0.36	0.01
Lens right	3.00 ± 0.56	2.13 ± 0.30	0.29	4.01 ± 0.70	2.58 ± 0.27	0.02

**Table 4:** Treatment times (in min) for “Patient 10” for both modalities.

	VMAT		HT	
	Morning Fraction [min]	Evening Fraction [min]	Morning Fraction [min]	Evening Fraction [min]
**Beam-on time**	51.00	49.78	30.88	30.88
**Image guidance**	64.80	84.83	43.87	48.40
**Couch adjustment**	20.85	28.27	0	0
**Patient rotation**	10.15	20.50	3.02	3.70
**Total treatment time**	146.8	183.38	77.76	82.98

**Table 5:** Average beam-on time (in min) for the upper body ± SD.

Beam-on Time	VMAT [min]	HT [min]
**8 Gy-group**	24.3 ± 6.4	16.7 ± 1.7
**12 Gy-group**	27.4 ± 6.2	16.7 ± 1.3

## Data Availability

All data relevant to this publication have been included in the manuscript’s body.

## Abbreviations

Total body irradiation (TBI); helical tomotherapy (HT); volumetric modulated arc therapy (VMAT); organs at risk (OAR); acute myeloid leukemia (AML); acute lymphoblastic leukemia (ALL); source-to-skin distance (SSD); computed tomography (CT); multi-leaf collimator (MLC); total marrow irradiation (TMI); total marrow and lymphoid irradiation (TMLI); mixed-phenotype acute leukemia (M-PAL); mast cell leukemia (MCL); treatment planning system (TPS); planning target volume (PTV); isocenters (IC); cone beam CT (CBCT); mega voltage CT (MVCT); homogeneity index (HI); D0 (decimal reduction dose); mean dose (Dmean); D95% (minimum absorbed dose that covers 95% of the volume of the PTV); D98% (minimum absorbed dose that covers 98% of the volume of the PTV); D50% (median absorbed dose in the PTV); D2% (minimum absorbed dose that covers 2% of the volume of the PTV).
